# Diet in Food Insecurity: A Mediator of Metabolic Health?

**DOI:** 10.1210/jendso/bvae062

**Published:** 2024-04-02

**Authors:** Lisa L Morselli, Rabia Amjad, Roland James, Tammy L Kindel, Anne E Kwitek, Joni S Williams, Justin L Grobe, Srividya Kidambi

**Affiliations:** Department of Medicine, Division of Endocrinology and Molecular Medicine, Medical College of Wisconsin, Milwaukee, WI 53226, USA; Cardiovascular Center, Medical College of Wisconsin, Milwaukee, WI 53226, USA; Center for Advancing Population Science, Medical College of Wisconsin, Milwaukee, WI 53226, USA; Department of Medicine, Division of Endocrinology and Molecular Medicine, Medical College of Wisconsin, Milwaukee, WI 53226, USA; Cardiovascular Center, Medical College of Wisconsin, Milwaukee, WI 53226, USA; Department of Surgery, Medical College of Wisconsin, Milwaukee, WI 53226, USA; Department of Physiology, Medical College of Wisconsin, Milwaukee, WI 53226, USA; Department of Biomedical Engineering, Medical College of Wisconsin, Milwaukee, WI 53226, USA; Linda T. and John A. Mellowes Center for Genomic Sciences and Precision Medicine, Medical College of Wisconsin, Milwaukee, WI 53226, USA; Cardiovascular Center, Medical College of Wisconsin, Milwaukee, WI 53226, USA; Center for Advancing Population Science, Medical College of Wisconsin, Milwaukee, WI 53226, USA; Department of Medicine, Medical College of Wisconsin, Milwaukee, WI 53226, USA; Cardiovascular Center, Medical College of Wisconsin, Milwaukee, WI 53226, USA; Department of Physiology, Medical College of Wisconsin, Milwaukee, WI 53226, USA; Department of Biomedical Engineering, Medical College of Wisconsin, Milwaukee, WI 53226, USA; Comprehensive Rodent Metabolic Phenotyping Core, Medical College of Wisconsin, Milwaukee, WI 53226, USA; Department of Medicine, Division of Endocrinology and Molecular Medicine, Medical College of Wisconsin, Milwaukee, WI 53226, USA; Cardiovascular Center, Medical College of Wisconsin, Milwaukee, WI 53226, USA

**Keywords:** food insecurity, energy intake, diet quality, adiposity, metabolic health

## Abstract

**Objective:**

Food insecurity (FI) is associated with poor metabolic health. It is assumed that energy intake and diet quality underlie this association. We tested the hypothesis that dietary factors (quantity and quality) mediate the association of FI with excess weight, waist circumference and glycemic control [glycohemoglobin (A1C)].

**Methods:**

A mediation analysis was performed on data from the National Health And Nutrition Examination Survey using FI as an independent variable; body mass index (BMI), waist circumference, and A1C as metabolic outcome variables and total energy intake, macronutrients, and diet quality measured by the Healthy Eating Index-2015 (HEI-2015) as potential mediators.

**Results:**

Despite a greater prevalence of obesity in participants experiencing FI, daily reported energy intake was similar in food-secure and -insecure subjects. In adjusted analyses of the overall cohort, none of the examined dietary factors mediated associations between FI and metabolic outcomes. In race-stratified analyses, total sugar consumption was a partial mediator of BMI in non-Hispanic Whites, while diet quality measures (HEI-2015 total score and added sugar subscore) were partial mediators of waist circumference and BMI, respectively, for those in the “other” ethnic group.

**Conclusion:**

Dietary factors are not the main factors underlying the association of FI with metabolic health. Future studies should investigate whether other social determinants of health commonly present in the context of FI play a role in this association.

Food insecurity (FI) is defined as the lack of consistent access to sufficient food for an active and healthy life [1]. Food insecurity was estimated to affect 17 million American households (12.7%) in 2022 [2], with a significantly higher prevalence in minorities [3].While prevalence of FI has consistently declined in the last decade, these most recent estimates are a significant increase from years preceding the COVID-19 pandemic [2].

Paradoxically, FI is associated with increased prevalence of obesity in high-income countries, especially in women [4]. Furthermore, individuals experiencing FI have an increased risk of several complications of obesity, such as metabolic syndrome [5], type 2 diabetes [6], and poor glycemic control in those with type 2 diabetes [7, 8].

Multiple studies have shown that FI is associated with poor diet quality, ie, a less healthful dietary pattern [9, 10]. Many energy-dense foods are cheaper in price and therefore easier to fit into a situation of financial constraint, often associated with FI [11]. These types of foods, usually highly processed, have been associated with higher daily energy (calorie) intake [12]. In addition, these foods are often of less favorable macronutrient composition, with higher fat and carbohydrate compared to protein content [13], which have been linked to adverse metabolic outcomes [14].

Poor diet quality has been hypothesized to contribute to the negative health consequences of food insecurity [15]. However, individuals with FI usually endure other social determinants of health such as lower personal and/or neighborhood socioeconomic status, precarious employment, short sleep and/or poor sleep quality, and distress, which have also been associated with increased adiposity and adverse metabolic health [10, 16-19]. Thus, it is possible that nondietary factors underlie this association.

Mediation analysis is a method to estimate whether an intervention or exposure affects an outcome through a potential causal mechanism [20]. As a first step to examine the factors underlying worse metabolic outcomes in FI, we performed a mediation analysis on data from the National Health and Nutrition Examination Survey (NHANES), a publicly available cross-sectional, nationally representative survey of Americans, which recorded measures of food insecurity as well as energy intake and diet quality. With this method, we tested the hypotheses that (1) total energy intake, (2) macronutrient intake, and (3) diet quality mediate the association of FI with excess weight, central adiposity, and glycemic control (Fig. 1). Because of the reported differences in the association of FI with obesity in women vs men [4], and because people living with FI are more often non-white [3], we planned a priori to stratify our analyses by sex as well as by race and ethnicity.

**Figure 1.**
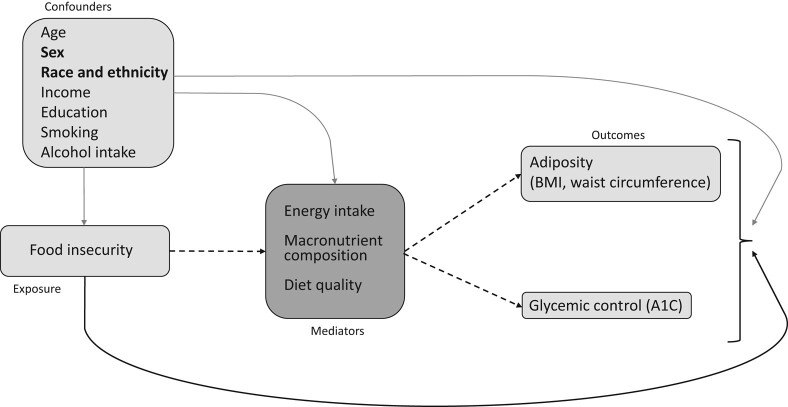
Directed acyclic graph for the assumed causal model: diet quantity and/or quality mediate the association of FI with adiposity (body mass index, waist circumference) and glycemic control (A1C). Full lines: direct effects. Dashed lines: natural indirect effects.

## Methods

### Subjects

We used data from the 2011-2012, 2013-2014, 2015-2016, and 2017-2018 cycles of NHANES [21]. Due to the COVID-19 pandemic, the 2019-2020 data was partial and not representative of the US population. Additionally, there is no appropriate weighting for these years. Therefore, the 2019-2020 cycle was not included in the analysis. Body composition measures were available from 2011-2012 onwards, thus prior cycles were not included.

The analysis was conducted on individuals who were at least 18 years old and answered the 10-item NHANES Food Security Survey Module, which is based on the 10-item US Department of Agriculture Adult Food Security Survey Module questionnaire [22]. Food security was defined by 2 or fewer affirmative answers and FI by 3 or more affirmative answers [7].

Age, race, and ethnicity (Hispanic, non-Hispanic Black, non-Hispanic White, other), income (monthly poverty level index ≤1.30, 1-31-1.85, >1.85), education (less than high school, high school graduate/GED, some college, college graduate), smoking status (never, current, former) and alcohol consumption were self-reported. Regarding alcohol intake, participants who responded they drank fewer than 12 alcohol-containing beverages in the last year were considered nondrinkers; those who reported drinking at least 12 alcohol-containing beverages but less than 5 drinks a day for men and 4 drinks a day for women were considered moderate drinkers; men reporting more than 5 drinks a day and women reporting more than 4 drinks a day were considered above-moderate drinkers.

### Measures of Metabolic Health

Weight, height, and waist circumference were measured by trained technicians. Obesity was defined as a body mass index (BMI) of 30 kg/m^2^ or greater and class III obesity as a BMI of 40 kg/m^2^ or greater. Measures of body composition are available from cycle 2011-2012 onward. However, some or all body composition data were missing in 54% of the participants; therefore, body composition was not included in the outcomes.

Glycohemoglobin (A1C) and fasting plasma glucose were measured in all participants. In cycles 2011-2012, 2013-2014, and 2015-2016, participants additionally underwent a 2-hour oral glucose tolerance test with plasma glucose assayed in fasting conditions and 2 hours after ingestion of 75 g of a glucose solution. Participants were also asked to report a diagnosis of diabetes mellitus (DM) and whether they took insulin or oral antidiabetes medications. Because the specificity of a self-reported diagnosis of DM is high but its sensitivity is variable [6, 23], DM was defined as self-reported DM and/or report of taking glucose-lowering drugs or fasting glucose ≥126 mg/dL or 2-hour oral glucose tolerance test glucose ≥200 mg/dL or A1C ≥ 6.5%, to reduce the risk of misclassification rates. Uncontrolled DM was defined as A1C ≥ 9%.

### Diet Composition

Dietary information was obtained from the What We Eat in America component of NHANES, which is based on 24-hour food recalls collected by trained interviewers on 2 separate days and provides estimates of nutrient intake [24]. Averages of both days were used for analysis.

### Diet Quality

Diet quality was captured with the Healthy Eating Index (HEI)-2015 [25]. This index uses a scoring system to evaluate a set of 13 dietary components that reflect the key recommendations in the *2015-2020 Dietary Guidelines for Americans*. The components include total fruit (cup equivalent/1000 kcal), whole fruit (cup equivalent/1000 kcal), total vegetables (cup equivalent/1000 kcal), greens and beans (cup equivalent/1000 kcal), whole grains (oz equivalents/1000 kcal), total dairy (cup equivalent/1000 kcal), total protein foods (oz equivalent/1000 kcal), seafood and plant protein (cup equivalent/1000 kcal), refined grains (oz equivalent/1000 kcal), fatty acids (ratio of polyunsaturated to monounsaturated fatty acids), added sugars (% of daily energy intake), saturated fats (% of daily energy intake), and sodium (g/1000 kcal). HEI-2015 scoring was performed using the HEI-2015 per person algorithm [26]. Total scores range from 0 to 100, with higher scores indicating higher diet quality. Of note, for subscores of less desirable components “added sugars,” “refined grains,” “sodium,” and “saturated fats,” higher scores represent *lower* consumption.

### Statistical Analysis

The demographic characteristics of the study population were summarized first by food security status using count and frequency for categorical variables and mean and SD for continuous variables (for normally distributed variables) or median and interquartile ranges (for nonnormally distributed variables). Next, means and SDs were calculated for mediators and HEI-2015 variables by food security status. Comparisons on continuous or categorical variables were performed by food security status using a *t*-test and Wald chi-square, respectively. Analyses were corrected for multiple comparisons with Bonferroni correction. The corresponding *P*-value thresholds for significance are reported in the tables.

**Figure 2.**
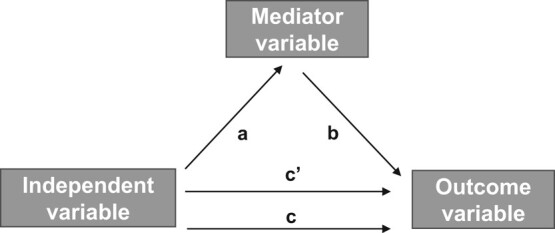
Mediation analysis with Baron and Kenny approach [27].

Lastly, a series of linear regression models (unadjusted and adjusted for age, sex, race, income, education, smoking, alcohol consumption for outcomes BMI and waist circumference, adjusted for same confounders and BMI for A1C) were performed for the mediation analysis using the Baron and Kenny approach [27] (Fig. 2):

- First, the association between outcome (Y) and independent variable (X) [FI] was analyzed (path c, direct effect).
- Second, the association between the mediator (M) and the independent variable (X) was tested (path a).
- Third, the association between outcome (Y) and mediator (M) was investigated (path b).
- Lastly, the association between outcome (Y) and independent variable (X) [FI] + mediator (M) was analyzed (path c”, indirect effect).

Models were repeated for each outcome and mediator pair. A variable was considered a significant *partial* mediator if the β coefficient for c” was at least 10% lower than for c and a *full* mediator if the β coefficient in c” at least 10% lower than for c *and* the association was no longer statistically significant. The analysis accounted for the complex survey design, and the significance of the result was evaluated at *P*-value <.05. Analysis was performed in SAS® software (SAS Institute, 2011) and R version 4.2.0 (2022-04-22 ucrt).

Associations of energy intake, specific macronutrients, and diet quality with metabolic health have been reported [14]. Therefore, prespecified potential mediators of the effect of FI on measures of excess weight, adiposity, and A1C included total energy intake (kcal/day), total fats (g/day), total carbohydrates (g/day), total sugars (g/day), total HEI-2015 score, and added sugar HEI-2015 subscore.

Subgroup analyses were conducted for the mediation analysis on A1C, restricting the analysis to participants with DM and uncontrolled DM.

To investigate whether sex or race and ethnicity differentially impact the results of the mediation analyses, race × outcome and sex × outcome interactions in the association of FI with each outcome were examined. Mediation analyses were stratified by race and ethnicity or sex only if a significant interaction was present.

This manuscript was prepared in compliance with the Guideline for Reporting Mediation Analysis [20]. A corresponding checklist is available in the supplemental data [28].

## Results

Demographic characteristics of the study cohort are presented in Table 1. This cohort included 23 146 adult (≥18 years old) participants (weighted N 234 428 206), of which 16.6% reported FI. Non-Hispanic Whites (NHW) represented the largest ethnic group among those with FI. However, non-Hispanic Blacks (NHB) and Hispanics were proportionally more represented in the FI group. NHB constituted 16.9% of the FI group despite representing 11.5% of the total cohort. Similarly, Hispanic participants represented only 6.3% of the total cohort but 11.2% of FI subjects. Subjects experiencing FI were also younger (*P* < .001) and more likely to be women (*P* = .03) than their food-secure counterparts. A greater proportion of participants experiencing FI reported low income (*P* < .001). Conversely, a greater proportion of participants reporting food security attended some college or graduated from college (*P* < .001). More individuals in the FI group were current smokers than those who were food-secure (*P* < .001). On the other hand, slightly fewer participants experiencing FI were more than moderate drinkers (*P* < .01).

**Table 1.** Demographics and clinical characteristics of study cohort

	All	Food security	Food insecurity	*P*-value (food security vs insecurity)
n	23 146	18 195	4915	
N	234 428 206	195 489 411	38 938 795	
Prevalence (%)	—	83.4	16.6	
Prevalence by data release cycle (N, %)				.23
2011-2012	—	48 752 214 (84.3)	9 097 944 (15.7)	
2013-2014	—	49 925 533 (85.0)	8 783 267 (15.0)	
2015-2016	—	48 010 200 (82.1)	10 498 138 (17.9)	
2017-2018	—	48 801 464 (82.2)	10 559 446 (17.8)	
Age (years)	47 ± 18	48 ± 18	42 ± 16	<.001
Women (%)	51.8	51.5	53.6	.01
Race (%)				<.001
Hispanic	6.3	5.3	11.2	
Non-Hispanic Black	11.5	10.4	16.9	
Non-Hispanic White	64.5	67.8	47.9	
Other	17.8	16.5	24	
Monthly poverty level index (%)				<.001
≤1.30	24.9	19.1	60.0	
1.31–1.85	11.9	11.6	16.6	
>1.85	59.1	69.3	23.4	
Education				<.001
Less than high school	14.8	12.0	29.4	
High school/GED	23.1	22.1	28.7	
Some college	31.8	31.5	33.3	
College graduate	30.1	34.5	8.6	
Smoking (%)				<.001
Never	57.4	59.4	47.6	
Current	18.6	15.5	33.9	
Former	24.0	25.1	18.6	
Alcohol consumption (%)				<.01
Nondrinker	17.9	18.9	21.8	
Moderate drinker*^a^*	18.9	20.7	19	
Above moderate drinker	55.8	60.4	59.2	
BMI (kg/m^2^)	29.2 ± 7.0	28.9 ± 6.8	30.4 ± 8.0	<.0001
BMI class				<.0001
<25 (%)	29.5	30.1	26.6	
25–29.9 (%)	32.2	33.1	27.6	
≥30 (%)	30.7	30.0	34.3	
≥40 (%)	7.6	6.9	11.4	
Waist circumference (cm)		99.1 ± 16.5	101.4 ± 18.3	<.001
Self-reported diabetes mellitus (%)				<.01
Yes	10.6	9.8	11.9	
No	89.4	90.2	88.1	
Taking diabetes medication				.06
Yes	9.2	9.0	10.3	
No	90.8	91	89.7	
Diabetes mellitus by clinical criteria (%)				<.0001
Yes	11.2	10.8	13.5	
No	88.8	89.2	86.5	
A1C (%)	5.6 ± 1.0	5.6 ± 0.9	5.8 ± 1.2	<.0001

Data expressed as mean ± SD for continuous variables.

Abbreviations: A1C, glycohemoglobin; BMI, body mass index; BP, blood pressure; GED, general education degree.

^a^Moderate drinker defined as more than 12 drinks in the last year but no more than 5 drinks per day in men and 4 drinks per day in women.

### Association Between FI and Dietary Factors

Energy and macronutrient intake are reported in Table 2. Reported total caloric intake was not different between food-secure and -insecure individuals (*P* = .93). In terms of macronutrients, individuals experiencing FI consumed more carbohydrates (*P* < .001) and total sugars (*P* < .001) and less total fats (*P* < .001) and protein (*P* < .0001). Similar results were obtained when expressing macronutrient consumption as percent of daily energy intake. They also consumed less sodium (*P* < .01) and potassium per day (*P* < .0001) compared to food-secure subjects. Of note, these differences, while statistically significant, were small and unlikely clinically significant. No differences between groups were seen in alcohol consumption expressed in grams/day.

**Table 2.** Energy intake and diet composition by food security status

	Food security	Food insecurity	*P*-value*^a^*
Energy intake (kcal/day)	2099 ± 814	2097 ± 983	.93
Carbohydrates			
(g/day)	246 ± 105	257 ± 127	<.0001
% total daily energy intake (%kcal)	48 ± 10	50 ± 10	<.0001
Total sugars (g/day)			
(g/day)	105 ± 62	117 ± 77	<0.0001
% total daily energy intake (%kcal)	21 ± 9	23 ± 10	<0.0001
Total fats			
(g/day)	83 ± 39	80 ± 42	<.001
% total daily energy intake (%kcal)	35 ± 7	34 ± 6	<.0001
Protein			
(g/day)	83 ± 36	78 ± 39	<.0001
% total daily energy intake (%kcal)	16 ± 4	15 ± 4	<.0001
Sodium (mg/day)	3498 ± 1484	3410 ± 1681	<.01
Potassium (mg/day)	2684 ± 1093	2435 ± 1158	<.0001
Alcohol (g/day)	10 ± 22	9 ± 27	.32

Data expressed as mean ± SD.

^a^Multiple comparison significance level *P* < .0125.

HEI-2015 scores (Table 3) were low on average in both groups, suggesting poor diet quality overall in this nationally representative cohort. Total HEI-2015 score was significantly lower in participants experiencing FI, indicating even worse dietary quality compared to individuals reporting food security (*P* < .0001). Similarly, all subscores were significantly lower with food insecurity (indicating lower consumption of fruit, vegetables, whole grains, and protein and higher consumption of refined grains and added sugars), except for sodium (higher score indicating lower consumption). Subscores for total dairy and saturated fats were not significantly different between the 2 groups.

**Table 3.** Diet quality by food security status

	Food security	Food insecurity	*P*-value*^a^*
Total HEI-2105 score	54 ± 14	49 ± 13	<.0001
Total fruit	2.34 ± 1.96	1.87 ± 1.97	<.0001
Whole fruit	2.56 ± 2.17	1.88 ± 2.15	<.0001
Total vegetables	3.28 ± 1.49	2.91 ± 1.55	<.0001
Greens and beans	2.03 ± 2.15	1.73 ± 2.11	<.0001
Whole grains	2.96 ± 3.22	2.22 ± 2.98	<.0001
Total dairy	5.31 ± 3.04	5.21 ± 3.12	.24
Total protein	4.48 ± 0.97	4.33 ± 1.14	<.0001
Seafood and plant protein	2.94 ± 2.13	2.34 ± 2.20	<.0001
Fatty acids	5.02 ± 3.37	4.65 ± 3.54	<.0001
Refined grains*^b^*	6.37 ± 3.37	5.71 ± 3.54	<.0001
Sodium*^b^*	4.03 ± 3.19	4.30 ± 3.36	<.001
Added sugars*^b^*	7.09 ± 3.09	6.00 ± 3.53	<.0001
Saturated fats*^b^*	5.73 ± 3.25	5.86 ± 3.28	.16

Data expressed as mean ± SD.

Abbreviation: HEI-2015, Healthy Eating Index-2015.

^a^Multiple comparison significance level *P* < .0003.

^*b*^Higher score represents lower consumption.

### Association Between FI and Excess Weight/Adiposity

Individuals experiencing FI had higher BMI overall than those with food security (Table 1) as well as a 25% higher prevalence of obesity, defined as BMI ≥30 kg/m^2^. Moreover, the prevalence of class III obesity, defined as BMI ≥40 kg/m^2^, was twice as high in the group experiencing FI compared to food-secure individuals. FI was also associated with a 2 cm higher waist circumference (*P* < .001).

### Diet Macronutrient Composition and Diet Quality as Mediators of the Association Between FI and Excess Weight/Adiposity

Unadjusted mediation analysis revealed that diet quality, measured as the total HEI-2015 score, was a partial mediator of the association of FI with BMI and with waist circumference. However, after adjustment for confounders, HEI-2015 was no longer a mediator of BMI or waist circumference (Table 4). Specifically, the combined associations of FI and HEI-2015 with BMI or waist circumference were still significant after adjustment for covariates, but the change in beta coefficient no longer met the criteria for HEI-2015 to be considered a mediator of these associations. No other dietary factor had a significant mediating effect (supplemental table [28]). In particular, total energy intake was not identified as a potential mediator (data not shown).

**Table 4.** Unadjusted and adjusted mediation analysis

	Unadjusted					Adjusted				
Subgroup	a β (95% CI)	b β (95% CI)	c β (95% CI)	c’ β (95% CI)	c” vs c Δβ	a β (95% CI)	b β (95% CI)	c β (95% CI)	c’ β (95% CI)	c” vs c Δβ
Outcome: BMI*^a^* Mediator: HEI-2015 (total score)										
Overall cohort	−5.10 (−5.83, −4.27) *P* < .0001	−0.06 (−0.07, −0.05) *P* < .0001	1.45 (1.02, 1.88) *P* < .0001	1.14 (0.66, 1.61) *P* < .0001	**−21%**	−1.66 (−2.33, −0.99) *P* < .0001	−0.07 (−0.08, −0.06) *P* < .0001	1.47 (1.03, 1.90) *P* < .0001	1.36 (0.91, 1.82) *P* < .0001	−7%
Hispanic	−1.70 (−3.15, −0.27) *P* = .02	−0.06 (−0.09, −0.03) *P* < .0001	1.40 (0.76, 2.04) *P* < .0001	1.27 (0.59, 1.94) *P* < .0001	−9%	NS	—	1.58 (0.74, 2.42) *P* < .001	—	n/a
NHB	−2.63 (−3.61, −1.66) *P* < .0001	—	NS	—	n/a	NS	—	NS	—	n/a
NHW	−6.76 (−7.86, −5.65) *P* < .0001	−6.76 (−7.86, −5.65) *P* < .0001	1.54 (0.78, 2.31) *P* < .001	1.11 (0.29, 1.93) *P* < .01	**−28%**	−2.49 (−3.51, −1.48) *P* < .0001	−0.08 (−0.09, −0.06) *P* < .0001	1.73 (0.98, 2.47) *P* < .0001	1.58 (0.81, 2.35) *P* < .001	−9%
Other	−4.87 (−5.96, −3.77) *P* < .0001	−0.08 (−0.10, −0.06) *P* < .0001	2.03 (1.35, 2.70) *P* < .0001	1.55 (0.83, 2.28) *P* < .0001	**−24%**	−2.23 (−3.33, −1.14) *P* < .001	−0.07 (−0.09, −0.05) *P* < .0001	1.38 (0.65, 2.12) *P* < .001	1.17 (0.39, 1.96) *P* < .01	**−15%**
Outcome: BMI*^a^* Mediator: Total sugar (g/day)										
Overall cohort	11.69 (8.10, 15.28) *P* < .0001	−0.003 (−0.01,−0.001) *P* = .01	1.45 (1.02, 1.88) *P* < .0001	1.48 (1.02, 1.94) *P* < .0001	+2%	4.80 (0.56, 9.04) *P* = .02	NS	−1.47 (1.03, 1.90) *P* < .0001	—	n/a
Hispanic	NS	—	1.40 (0.76, 2.04) *P* < .0001	—	n/a	NS	—	1.58 (0.74, 2.42) *P* < .001	—	n/a
NHB	NS	—	NS	—	n/a	NS	—	NS	—	n/a
NHW	16.92 (10.62, 23.21) *P* < .0001	−0.004 (−0.01,−0.001) *P* < .01	1.54 (0.78, 2.31) *P* < .0001	1.63 (0.85, 2.41) *P* < .0001	+6%	6.97 (0.19, 13.75) *P* = .04	−0.004 (−0.01, −0.0004) *P* = .03	1.73 (0.98, 2.47) *P* < .0001	1.29 (0.51, 2.09) *P* < .01	**−25%**
Other	9.62 (4.40, 14.84) *P* < .001	0.01 (0.003, 0.01) *P* < .01	2.03 (1.35, 2.70) *P* < .0001	1.85 (1.13, 2.58) *P* < .0001	−9%	NS	—	1.39 (0.65, 2.12) *P* < .001	—	n/a
Outcome: BMI*^a^* Mediator: Added sugar (HEI-2015 subscore)										
Overall cohort	−1.08 (−1.27, −0.90) *P* < .0001	−0.06 (−0.11, −0.01) *P* = .02	1.45 (1.02, 1.88) *P* < .0001	1.40 (0.94, 1.85) *P* < .0001	−3%	−0.38 (−0.58, −0.17) *P* < .001	−0.05 (−0.10, −0.004) *P* = .03	1.47 (1.03, 1.90) *P* < .0001	1.46 (1.01, 1.91) *P* < .0001	−1%
Hispanic	−0.69 (−1.05, −0.32) *P* < .001	NS	1.40 (0.76, 2.04) *P* < .0001	—	n/a	NS	—	1.58 (0.74, 2.42) *P* < .001	—	n/a
NHB	NS	—	NS	—	n/a	NS	—	NS	—	n/a
NHW	−1.50 (−1.83, −1.16) *P* < .0001	NS	1.54 (0.78, 2.31) *P* < .0001	—	n/a	−0.50 (−0.84, −0.15) *P* < .01	NS	1.73 (1.01, 2.54) *P* < .0001	—	n/a
Other	−1.02 (−1.34, −0.71) *P* < .0001	−0.28 (−0.37, −0.19) *P* < .0001	2.03 (1.35, 2.70) *P* < .0001	1.66 (0.93, 2.39) *P* < .0001	**−22%**	−0.63 (−0.92, −0.29) *P* < .0001	−0.22 (−0.31, −0.12) *P* < .0001	1.38 (0.65, 2.12) *P* < .001	1.20 (0.39, 2.0) *P* < .01	**−13%**
Outcome: Waist circumference*^a^* Mediator: HEI-2015 (total score)										
Overall cohort	−5.10 (−5.83, −4.27) *P* < .0001	−0.15 (−0.17, −0.12) *P* < .0001	2.25 (1.19, 3.31) *P* < .0001	1.52 (0.44, 2.62) *P* = .01	**−32%**	−1.66 (−2.33, −0.99) *P* < .0001	−0.18 (−0.21, −0.16) *P* < .0001	3.24 (2.13, 4.34) *P* < .0001	2.97 (1.81, 4.12) *P* < .0001	−8%
Hispanic	−1.70 (−3.15, −0.27) *P* = .02	−0.014 (−0.21, −0.08) *P* < .0001	3.03 (1.23, 4.84) *P* < .01	2.67 (0.91, 4.45) *P* < .01	**−12%**	NS	—	3.54 (1.38, 5.70) *P* < .01	—	n/a
NHB	−2.63 (−3.61, −1.66) *P* < .0001	−	NS	−	n/a	NS	—	NS	—	n/a
NHW	−6.76 (−7.86, −5.65) *P* < .0001	−0.17 (−0.20, −0.13) *P* < .0001	3.13 (1.29, 4.87) *P* < .04	2.03 (0.12, 3.94) *P* = .04	**−35%**	−2.49 (−3.51, −1.48) *P* < .0001	−0.20 (−0.24, −0.17) *P* < .0001	4.18 (2.38, 5.98) *P* < .0001	3.76 (1.90, 5.61) *P* < .001	**−10%**
Other	−4.87 (−5.96, −3.77) *P* < .0001	−0.17 (−0.22, −0.12) *P* < .0001	3.80 (2.19, 5.42) *P* < .0001	2.96 (1.28, 4.64) *P* < .001	**−22%**	−2.23 (−3.33, −1.14) *P* < .0001	−0.15 (−0.20, −0.11) *P* < .0001	2.85 (1.05, 4.66) *P* < .01	2.36 (0.43, 4.30) *P* = .02	**−17%**
Outcome: Hemoglobin A1C*^b^* Mediator: Total sugar (g/day)										
Subjects with A1C ≥ 9	27.77 (1.53, 54.02) *P* < .04	0.003 (0.001, 0.005) *P* < .01	0.43 (0.19, 0.67) *P* < .001	0.24 (−0.01, 0.49) *P* = .06	**−44%**	29.97 (9.10 50.83) *P* < .01	—	NS	—	n/a
Outcome: Hemoglobin A1C*^b^* Mediator: Added sugar (HEI-2015 subscore)										
Subjects with A1C ≥ 9	−1.07 (−1.88, −0.27) *P* < .01	−0.09 (−0.13, −0.04) *P* < .01	0.43 (0.19, 0.67) *P* < .001	0.23 (−0.02, 0.47) *P* = .07	**−47%**	−0.90 (−1.70, −0.10) *P* = .03	—	NS	—	n/a

A mediator was considered significant if the β coefficient of the c” relationship was lower than the β coefficient of the c relationship by 10% or more. Values in bold indicate change in β coefficient meeting criteria for significant mediator.

Path a association between FI and potential mediator; path b association between potential mediator and outcome; path c association between FI and outcome; path c” association between [FI + potential mediator] and outcome.

Abbreviations: A1C, glycohemoglobin; BP, blood pressure; CI, confidence interval; HEI-2015, Healthy Eating Index-2015; n/a, not applicable; NHB, non-Hispanic Black; NHW, non-Hispanic White; NS, not significant.

^*a*^Models adjusted for age, sex, race, income, education, smoking, alcohol consumption.

^*b*^Models adjusted for age, sex, body mass index, race, income, education, smoking, alcohol consumption.

Significant sex × BMI and sex × waist circumference interactions were present. However, when models were stratified by sex, all 3 paths (a, b, c) were not significant for all the outcome-mediator combinations. Therefore, mediation analysis was not stratified by sex.

Significant BMI × race/ethnicity interactions were found for total HEI-2015 score, as well as total sugar consumption and added sugars HEI-2015 subscore. In unadjusted analyses, total HEI-2015 was a significant partial mediator of the association of FI with BMI in NHW and the Other group, but not in NHB. After adjustment for confounders, total HEI-2015 remained a significant mediator of BMI in the Other group only (Table 4). In the adjusted analysis, total sugar intake was a significant partial mediator of BMI only in NHW, while added sugar HEI-2015 subscore was a partial mediator of BMI only in the Other group (Table 4).

A significant waist circumference × race/ethnicity interaction was also present; therefore analysis was further stratified for this outcome. In unadjusted analyses, total HEI-2015 score was a significant partial mediator of the association between FI and waist circumference in NHW, Hispanics, and Others but not NHB. In adjusted analyses, total HEI-2015 score remained a significant partial mediator in NHW and the Other ethnic group (Table 4).

### Association of FI and Glycemic Control

In the full cohort, 11.9% of subjects experiencing FI reported a diagnosis of DM, 10.9% reported taking glucose-lowering medications, and 13.5% had DM by clinical criteria vs 9.8%, 9%, and 10.8% of subjects with food security, respectively **(**Table 1). Overall, A1C was higher in those reporting FI vs participants with food security (*P* < .0001). Demographics and clinical characteristics of the study population identified as having DM are presented in Table 5 and were similar to the overall cohort in terms of age, sex, race, income, and education. Among subjects with DM, 25% reported experiencing FI. No difference by food security status was seen in the proportion of individuals on glucose-lowering agents.

**Table 5.** Demographics and glycemic control in subjects with diabetes mellitus

	All	Food security	Food insecurity	*P*-value (food security vs insecurity)
n	4273	3220	1053	
N	33 754 624	27 275 739	6 478 885	
%		80.8	19.2	
Age (years)	59.3 ± 14.0	60.3 ± 13.8	55.0 ± 14.0	<.0001
Sex (%)				<.0001
Women	48.7	46.7	57.2	
Men	51.3	53.3	42.8	
Race (%)				<.0001
Hispanic	6.2	4.8	11.9	
Non-Hispanic Black	14.3	13.2	19.0	
Non-Hispanic White	59.6	63.5	43.2	
Other	19.9	18.4	25.9	
Monthly poverty level index (%)				<.0001
≤1.30	28.9	20.9	62.3	
1.31–1.85	14.4	14.0	16.2	
>1.85	56.7	65.1	21.5	
Education				<.0001
Less than high school	20.9	17.3	36.2	
High school/GED	25.5	25.0	27.8	
Some college	31.8	32.5	29.0	
College graduate	21.7	25.2	7.0	
A1C (%)	7.1 ± 1.6	7.1 ± 1.5	7.5 ± 2.0	<.0001
Uncontrolled diabetes mellitus (%)				<.0001
Yes	12.3	10.8	18.7	
No	87.7	89.2	81.3	
Taking diabetes medications (%)				.26
Yes	64.0	64.6	61.8	
No	36.0	35.4	38.2	
A1C in subjects taking diabetes medications (%)	7.4 ± 1.6	7.3 ± 1.5	7.8 ± 1.9	<.0001

Data expressed as mean ± SD for continuous variables.

Abbreviations: A1C, glycohemoglobin; GED, general education degree.

A1C was 0.5% higher in those with FI, whether we considered the whole cohort or only those taking medications for diabetes (*P* < .0001 for both comparisons). The proportion of subjects with uncontrolled diabetes was 73% higher among those with FI (*P* < .0001).

### Diet Composition and Quality as Mediators of the Association Between FI and Glycemic Control

No mediation effect of any of the prespecified dietary factors was found in the study cohort overall or in the cohort including all subjects with DM (supplemental table [28]). In those with *uncontrolled* diabetes, total sugar consumption and the added sugar HEI-2015 subscore both were full mediators of the association between FI and A1C in unadjusted models but not after controlling for confounders (Table 4). In particular, the association between FI and A1C was no longer significant after adding income and education into the model. No significant interaction of A1C by sex or race and ethnicity was identified in this subgroup, and the mediation analysis was not stratified further.

## Discussion

In this study, contrary to our hypothesis, we found that, despite lower diet quality in individuals experiencing FI, neither energy intake nor macronutrient composition or diet quality mediated the association of FI with measures of excess weight (BMI) and central adiposity (waist circumference). Further, dietary factors did not mediate the association between FI and glycemic control based on A1C. Our findings expand on prior reports of an association between FI and poor diet quality [9] on one hand, and FI and adverse metabolic health on the other [4-8, 29, 30]. However, the results also suggest that diet quality in FI may not be as central to the association with poor metabolic health as previously thought and that other factors, possibly including other social determinants of health often incurred by individuals facing FI [18], may be playing a role.

Several “Food is Medicine” interventions have been conducted in recent years and have been shown to improve diet quality as well as food security [31]. While many of these trials also reported some improvement in health outcomes, including weight, BMI, A1C, and blood pressure, most were limited by small number of participants and pre-post design. Notably, among the 6 trials that included a control group and enrolled participants with FI [32-34], the impact of these interventions on weight and/or A1C was null or limited, which is in line with our results.

Contrary to our expectations, energy intake was similar between those with and without FI, and energy intake was not a mediating factor of the association of FI with excess weight or adiposity. This was surprising since highly processed foods, which a majority of individuals experiencing FI report consuming [35], have been associated with higher calorie intake and weight gain [12]. A few studies conducted on prior NHANES cohorts that reported energy intake also found no difference by food security status [9, 36, 37]. Dietary information collected in NHANES is based on self-report, which may not be accurate due to underreporting [38]. However, 24-hour food recalls administered by trained interviewers are considered an adequate structured method to collect information about dietary habits in epidemiologic studies, and the Automated Multiple-Pass Method used in NHANES has been shown to reduce underreporting bias, although more so in individuals with normal weight than with obesity [39]. Our findings may seem at odds with results of experimental studies comparing subjects with and without FI given ad libitum food access, which show *greater* food intake in participants experiencing FI [40, 41]. In the study by Stinson et al [40], subjects with FI observed for 72 hours in a vending machine paradigm scored higher than subjects without FI on the disinhibition scale of the Three Factor Eating Questionnaire and on the Binge Eating Scale. Thus, when food is *accessible*, individuals experiencing FI may eat more than their daily calorie needs.

In addition, despite similar reported energy intake, individuals with FI had a higher BMI and waist circumference than food-secure individuals. It is tempting to speculate that FI triggers mechanisms that support greater energy efficiency, ie, greater ability to store calories and nutrients as body fat, consistent with the “insurance hypothesis,” which posits that more efficient storage of body fat is an adaptive response to periods of low food availability [4]. Indeed, energy balance was assessed by whole-room indirect calorimetry in healthy individuals with and without FI matched for age, sex, BMI, and race and ethnicity [42]. Lower lipid oxidation rates were observed in those with FI, which is in line with the insurance hypothesis. Alternatively, individuals with FI could be less active than those who are food-secure and ultimately have a positive energy balance despite a similar energy intake. However, physical activity was measured by accelerometry in other NHANES cohorts (2003-2006 cycles) and no differences by food security status were found in adults in terms of objectively measured sedentary time or time spent in light and moderate physical activity [43]. Another possible explanation for these findings is that, although average protein intake was adequate, there was a large interindividual variability and some participants reported low protein intake. As low protein diets are associated with a smaller thermic effect of food, this may, over time, contribute to positive energy balance [44].

Although FI and several dietary factors were associated with A1C, we did not find any mediation effect of these dietary factors on A1C in the cohort overall or in a sensitivity analysis including all participants with DM. In those with uncontrolled DM, the association between FI and A1C became insignificant after introducing income and education in the models, supporting an impact of socioeconomic factors on glycemic control in this group, possibly due to competing priorities or barriers to accessing quality care [45].

In our analysis, there was no mediation effect of diet quality on BMI or waist circumference in NHB. In fact, neither diet quality nor FI were associated with BMI or waist circumference in this subgroup. On the other hand, diet quality partially mediated the effect of FI on waist circumference in NHW, with a trend for a partial mediation effect on BMI in this group. These results are concordant with findings an analysis of the National Health Interview Survey showing that the odds of being overweight or suffering from obesity in the setting of FI were increased in White and Hispanic women but not in Black women [30]. Additionally, others have reported either higher diet quality in Blacks with FI compared with Whites with FI [46] or no association between FI and diet quality in NHB [47]. It is possible that, because of their long history of hardship and discrimination, Blacks have developed better coping strategies to mitigate the effects of FI on diet quality [46]. A mediation effect of diet quality on BMI and waist circumference was also seen in the Other ethnic group. Given the heterogeneous composition of this group, it is difficult to draw any conclusion.

This study has several strengths, including large sample size, a nationally representative cohort, and rigorous and standardized data collection methods. We must, however, acknowledge several limitations. First, given the cross-sectional nature of the analysis, no inference on causality can be drawn. Second, as discussed earlier, our results could be affected by underreporting of dietary intakes. Third, while food security questionnaires used in NHANES ask participants to reflect on the previous 12 months, food recalls were obtained on 2 days only. Given the cyclic nature of FI [48], it cannot be ruled out that food-insecure participants were in a period of relative food security when interviewed about their eating habits. Fourth, we did not include body composition measures in our analysis, due to a large percentage (>50%) of missing data. Fifth, we did not control our analyses for self-reported physical activity; however, as mentioned, objective physical activity was not found to differ by FI status in the 2014-2015 cycles of NHANES [43]. Lastly, we did not include information on additional social determinants of health other than income and education.

In conclusion, diet quality and energy intake do not appear to mediate the associations between FI and measures of metabolic health. Future work should investigate the role of other social determinants of health in this association. Additionally, individuals facing FI have greater adiposity despite similar reported energy intake. The impact of FI on the physiology of energy balance needs to be better defined.

## Data Availability

Original data generated and analyzed during this study are included in this published article or in the data repositories listed in References.
